# Supplementary figures and images for: Toxoplasma gondii infection of neurons alters the production and content of extracellular vesicles directing astrocyte phenotype and contributing to the loss of GLT-1 in the infected brain
Source: PLoS Pathog. 2025 Jun 16;21(6):e1012733. doi: 10.1371/journal.ppat.1012733 (PMC12193631; doi:10.1371/journal.ppat.1012733)

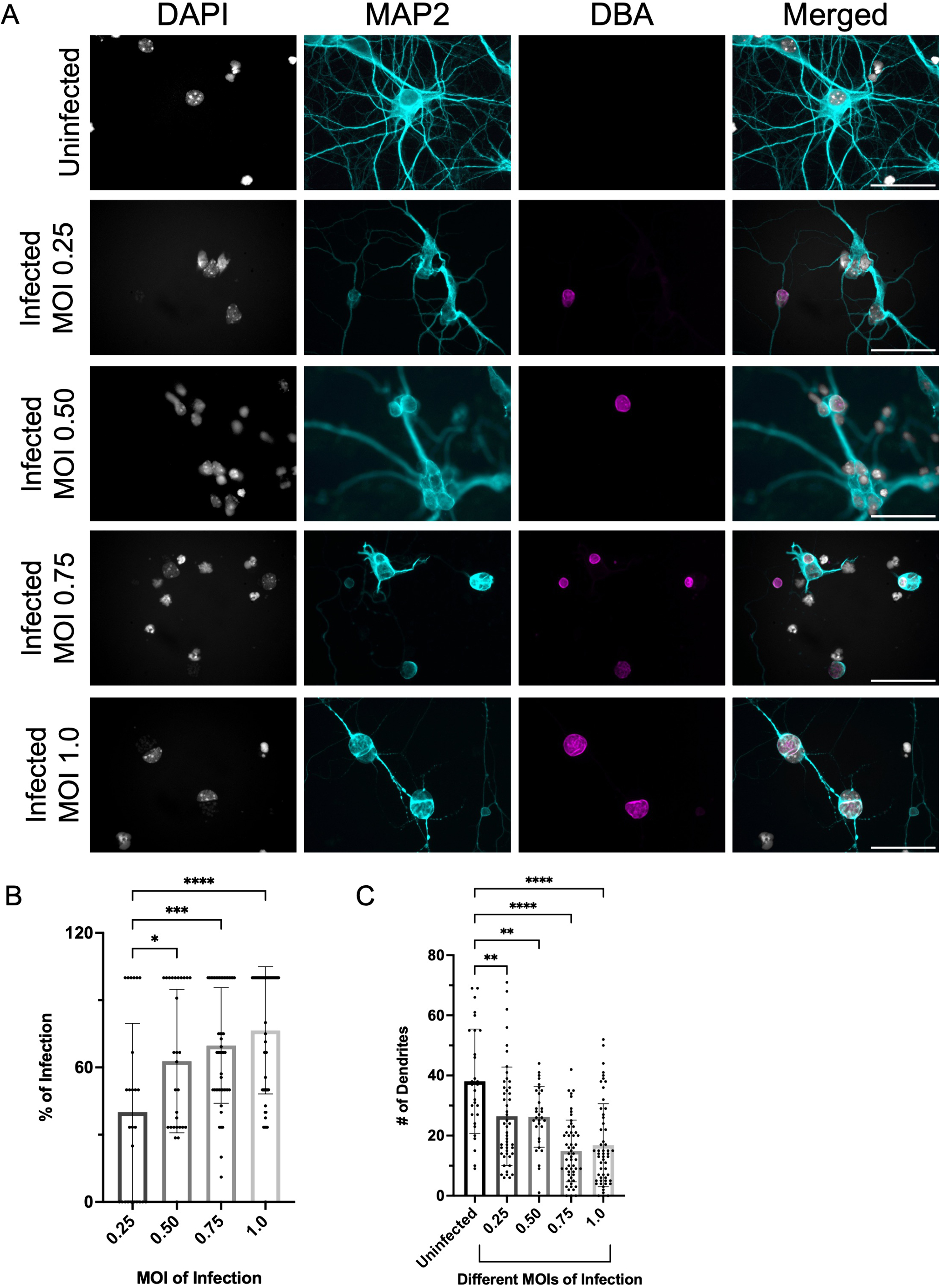

Supplement: S1 Fig — For quantifications “n” represents a single cell throughout multiple experiments. (A) Images taken three days post infection. DAPI stains the nucleus, MAP2 stains the perikarya and dendrites on neurons, DBA stains the sugars of the cyst wall. In all four different infected MOIs there is overlap of the cyst and neuron. Scale bar at 50 µm. (B) Quantification of the percentage of infected neurons. The number of cysts and neuronal nuclei were counted and the percentage of infection was calculated (One-way ANOVA, n (0.25) = 26, n (0.50) = 29, n (0.75) = 49, n (1.0) = 55, 0.25 vs. 0.50 p value = 0.0314, 0.25 vs. 0.75 p value = 0.0005, 0.25 vs. 1.0 p value = < 0.0001, 0.50 vs. 0.75 p value = 0.7621, 0.50 vs. 1.0 p value = 0.2045, 0.75 vs. 1.0 p value = 0.6699). (C) Quantification of the number of dendrites from neurons using Neurolucida. The number of dendrites for each of the infected neuronal groups was compared to the uninfected control group (One-way ANOVA, n (Uninfected) = 29, n (0.25) = 49, n (0.50) = 31, n (0.75) = 54, n (1.0) = 55, Uninfected vs. 0.25 p value = 0.0014, Uninfected vs. 0.50 p value = 0.0036, Uninfected vs. 0.75 p value = < 0.0001, Uninfected vs. 1.0 p value = < 0.0001). (TIF) [file ppat.1012733.s001.tif]

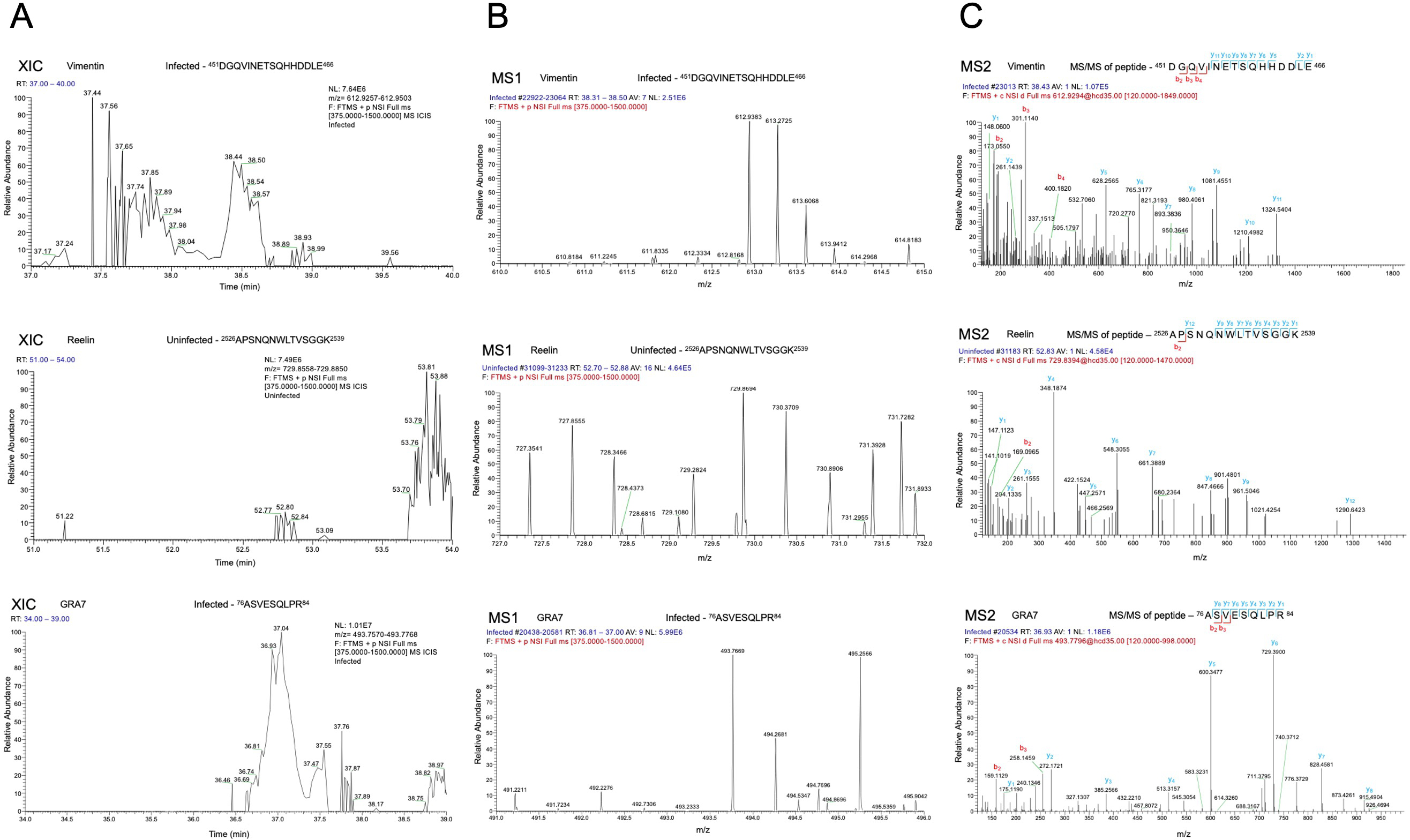

Supplement: S2 Fig — (A) Extracted ion chromatography (XIC) for the three different significantly regulated proteins. Vimentin (Vim) is upregulated during infection. Reelin (Reln) is downregulated during infection. GRA7 is found in EVs from infected neurons. (B) MS1 (first pass) graphs for the peptide sequences for each of the three different proteins. (C) MS2 (second pass) for the peptide sequences along with the y and b ions to confirm sequence. (TIF) [file ppat.1012733.s002.tif]

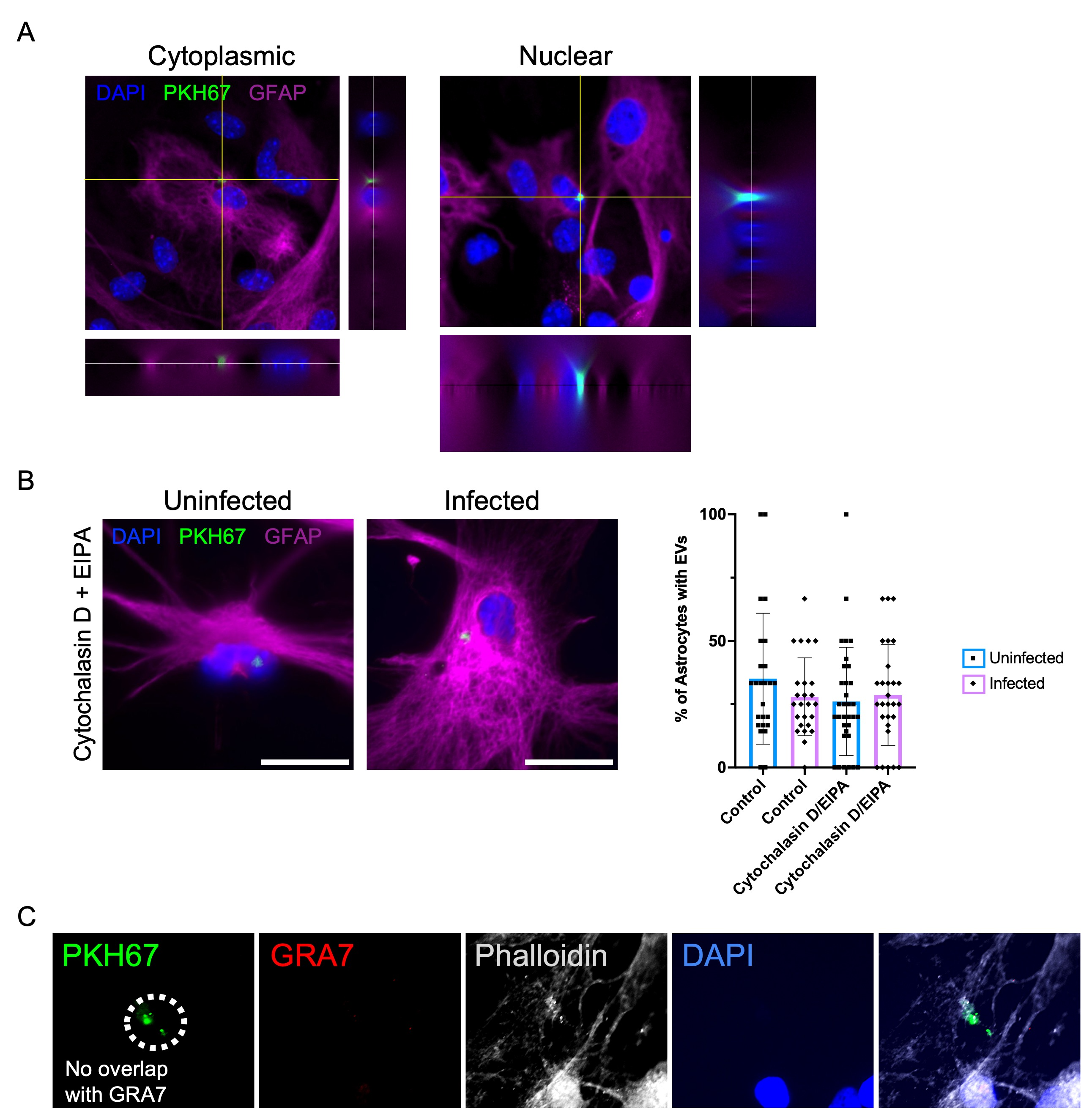

Supplement: S3 Fig — (A) Orthogonal view of the z-stacks highlighting astrocyte (GFAP) uptake of EVs (PKH67) in the XZ and YZ planes for both groups. (B) Fluorescence microscopy of astrocytes after the addition of uptake blockers and EVs from uninfected and T. gondii infected neurons. Scale bar: 20 µm. Quantification of the percentage of astrocytes that contain EVs. “n” represents a single cell across multiple experiments (Unpaired t-test, n (Uninfected Control) = 25, n (Infected Control) = 25, n (Uninfected Cytochalasin D/EIPA) = 33, n (Infected Cytochalasin D/EIPA) = 28, Uninfected Control vs. Uninfected Cytochalasin D/EIPA p = 0.1531, Infected Control vs. Infected Cytochalasin D/EIPA p = 0.8922). (C) Not all EVs from infected neurons (PKH67) are GRA7 positive. DAPI stains the nucleus, PKH67 stains the EVs, GRA7 stains the internalized GRA7 proteins, Phalloidin stains the actin filaments of the astrocyte. (TIFF) [file ppat.1012733.s003.tiff]

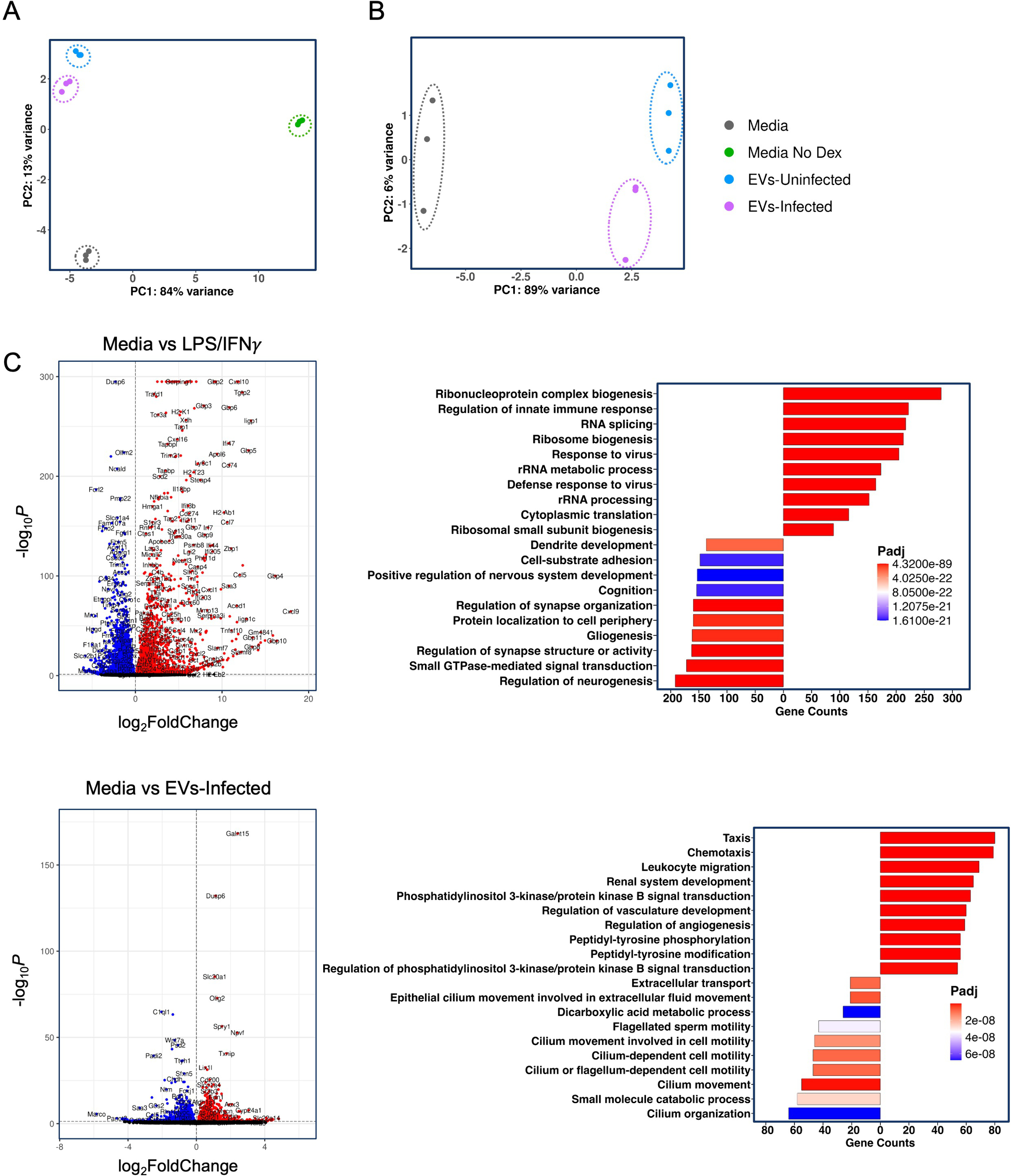

Supplement: S4 Fig — (A) PCA of astrocytes after 24 hours with the addition of cell culture media (gray), cell culture media without dexamethasone (green), EVs from uninfected neurons (blue) and EVs from infected neurons (purple). (B) PCA plot excluding cell culture media without dexamethasone values. (C) Volcano plot and GO analysis of DEGs comparing astrocytes RNA after the addition of media, LPS/IFNγ, and EVs from infected neurons. For the volcano plot anything above -log10(0.05) was considered significant. Upregulated genes are shown in red, downregulated shown in blue, and no change in black. For GO analysis bar graphs to the right of 0 indicate upregulated GO terms and to the left represent downregulated GO terms. Bar graphs are colored accorded to adjusted p value (padj). (TIF) [file ppat.1012733.s004.tif]

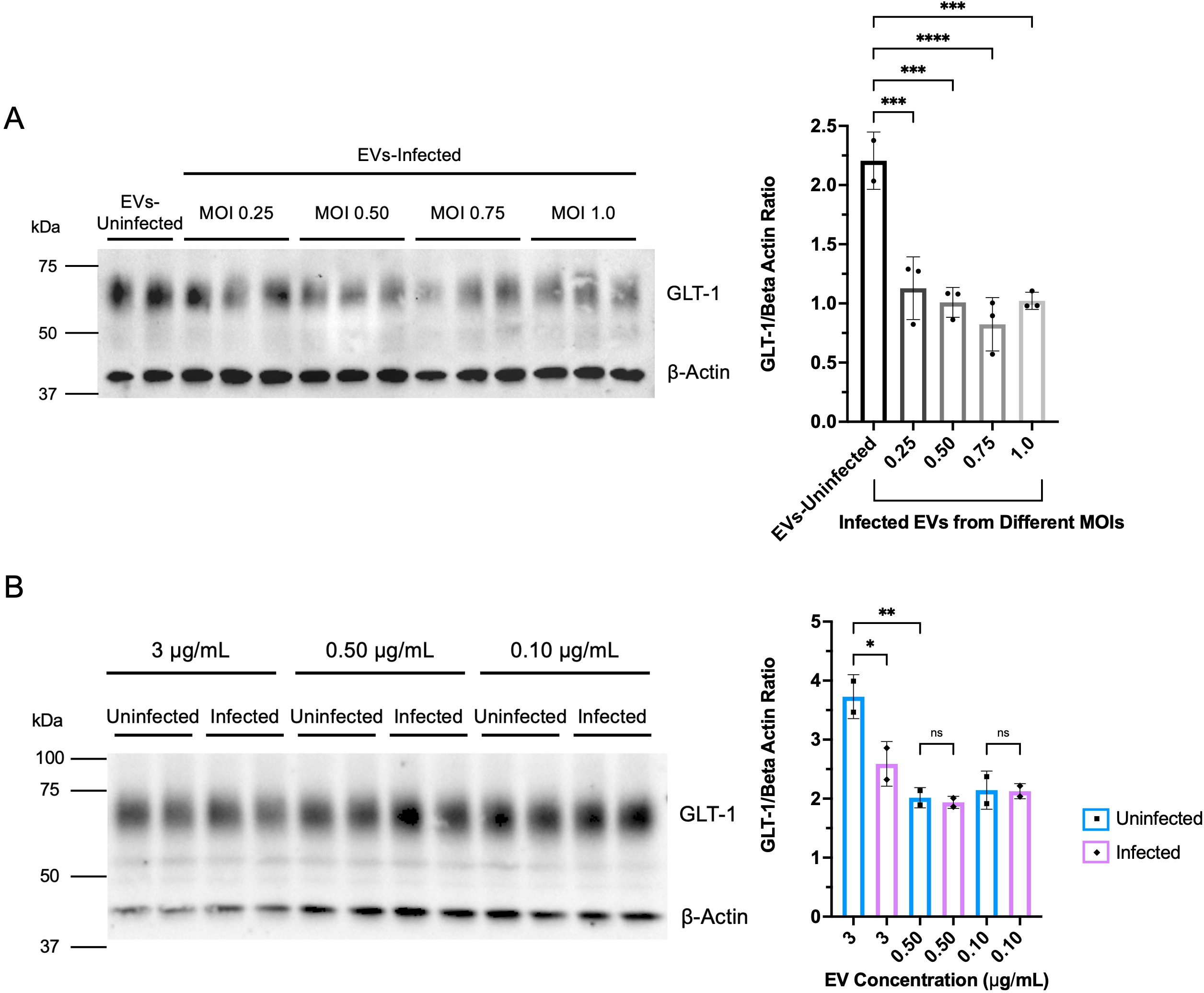

Supplement: S5 Fig — (A) Astrocytes with the addition of EVs from uninfected neurons were used as a control to compare with the remaining sample. β-Actin (~42 kDa) was utilized as a loading control. GLT-1 (~62 kDa) was present in all samples with different intensities. Image of gel was taken 166.2 seconds after exposure. Quantification of Western Blot bands using Fiji (ImageJ). The area of the bands in pixels was measured for both β-Actin and GLT-1. A ratio of the pixels (GLT-1/β-Actin) was taken and plotted (One-way ANOVA, n (EVs-Uninfected) = 2, n (EVs-Infected MOI 0.25) = 3, n (EVs-Infected MOI 0.50) = 3, n (EVs-Infected MOI 0.75) = 3, n (EVs-Infected MOI 1.0) = 3, EVs-Uninfected vs. EVs-Infected MOI 0.25 p value = 0.0006, EVs-Uninfected vs. EVs-Infected MOI 0.50 p value = 0.0003, EVs-Uninfected vs. EVs-Infected MOI 0.75 = < 0.0001, EVs-Uninfected vs. EVs-Infected MOI 1.0 p value = 0.0003). (B) Western blot for GLT-1 protein expression after the addition of different EV concentrations from uninfected and infected EVs. β-Actin (~42 kDa) was utilized as a loading control. GLT-1 (~62 kDA) was present in all samples with different intensities. Image of gel was taken 29.9 seconds after exposure. Quantification of Western Blot bands using Fiji (ImageJ). The area of the bands in pixels was measured for both β-Actin and GLT-1. A ratio of the pixels (GLT-1/β-Actin) was taken and plotted (One-way ANOVA, n (EVs-Uninfected 3 µg/mL) = 2, n (EVs-Infected 3 µg/mL) = 2, n (EVs-Uninfected 0.50 µg/mL) = 2, n (EVs-Infected 0.50 µg/mL) = 2, n (EVs-Uninfected 0.10 µg/mL) = 2, n (EVs-Infected 0.10 µg/mL) = 2, Uninfected 3 μg/mL vs. Infected 3 μg/mL p value = 0.0403, Uninfected 3 μg/mL vs. Uninfected 0.50 μg/mL p value = 0.0057, Uninfected 3 μg/mL vs. Infected 0.50 μg/mL p value = 0.0045, Uninfected 3 μg/mL vs. Uninfected 0.10 μg/mL p value = 0.0085, Uninfected 3 μg/mL vs. Infected 0.10 μg/mL p value = 0.0080, Infected 3 μg/mL vs. Uninfected 0.50 μg/mL p value = 0.3896, Infected 3 μg/mL vs. Infected 0.50 μ [file ppat.1012733.s005.tif]

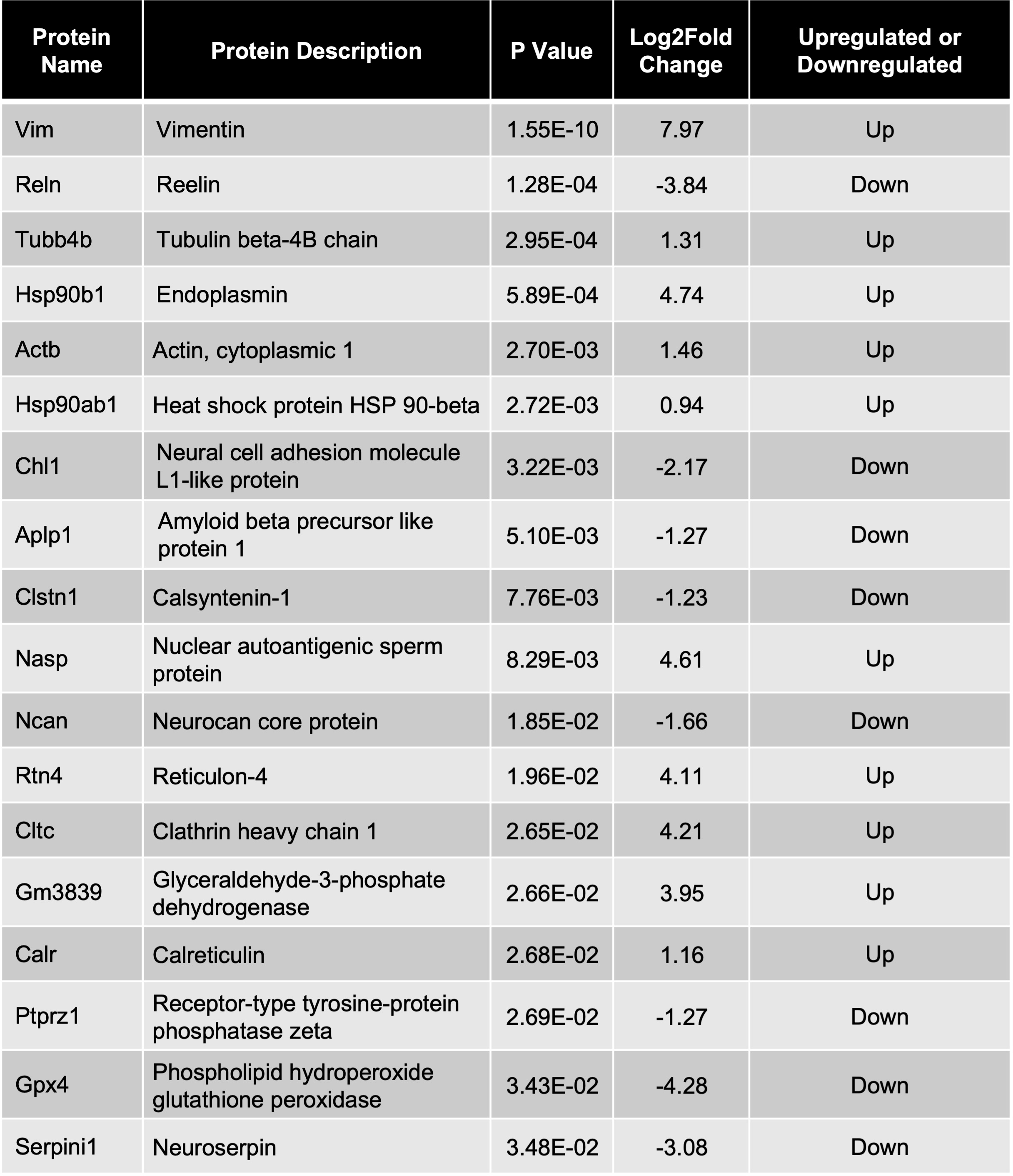

Supplement: S1 Table — Upregulated proteins are more involved in a stress or interferon response and structural support. Downregulated proteins are more involved with neuronal growth and migration. (TIF) [file ppat.1012733.s006.tif]

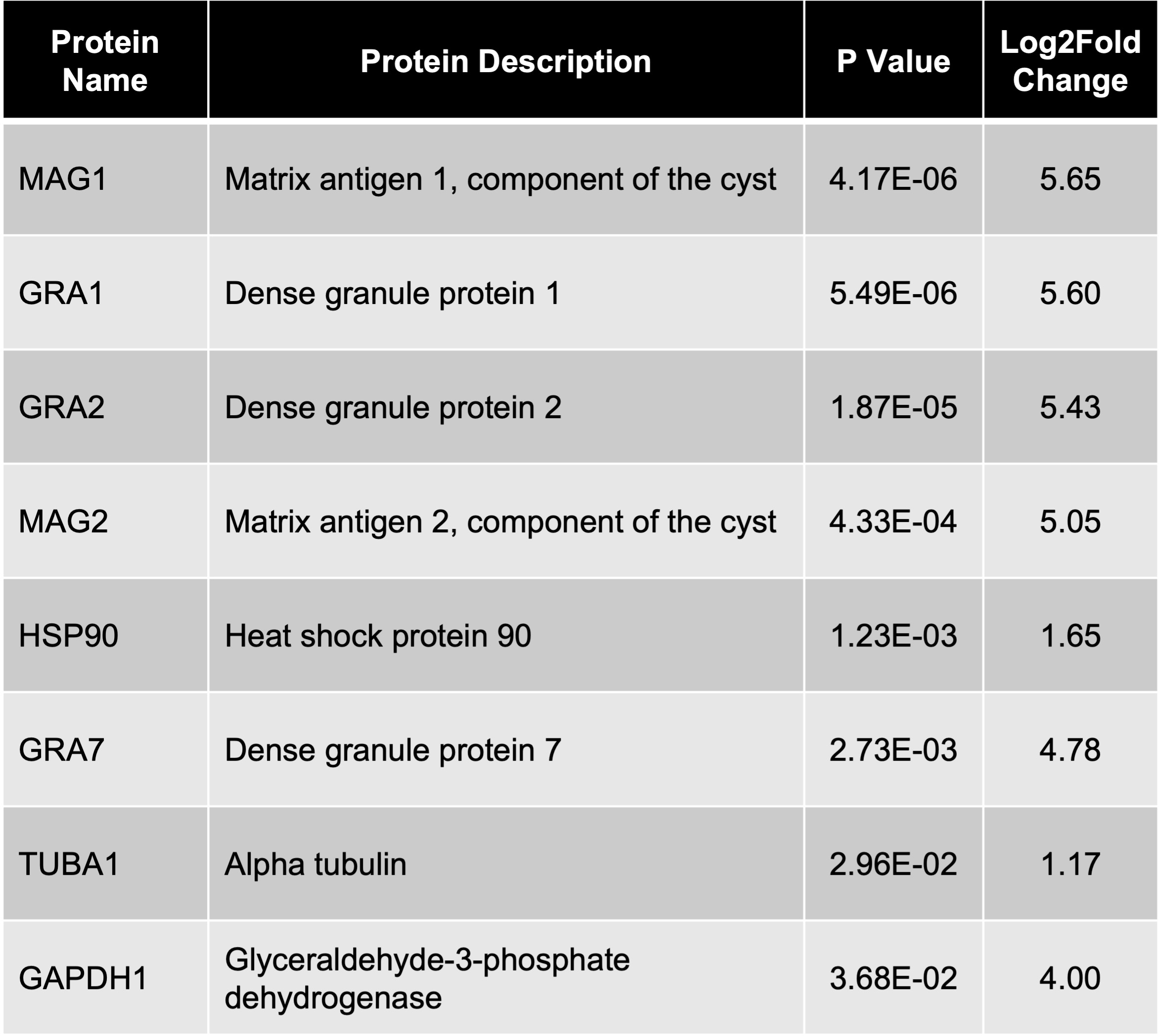

Supplement: S2 Table — Upregulated proteins consistent of parasite specific dense granules (GRAs) and matrix antigen proteins (MAGs) involved in cyst formation, along with some housekeeping proteins. (TIF) [file ppat.1012733.s007.tif]

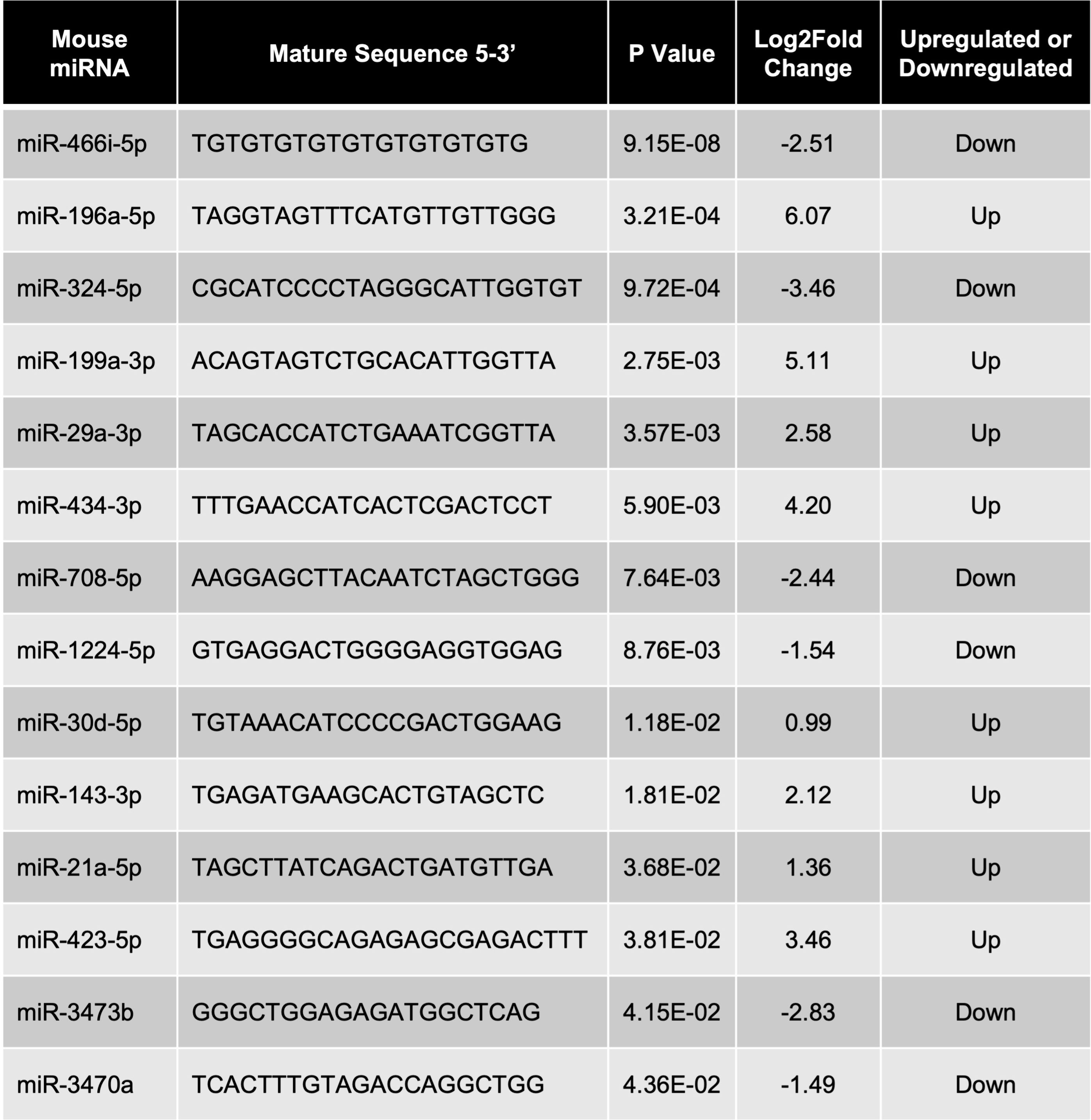

Supplement: S3 Table — Upregulated miRNA are more related to an anti-inflammatory response. Downregulated miRNA are more involved in a pro-inflammatory response. (TIF) [file ppat.1012733.s008.tif]

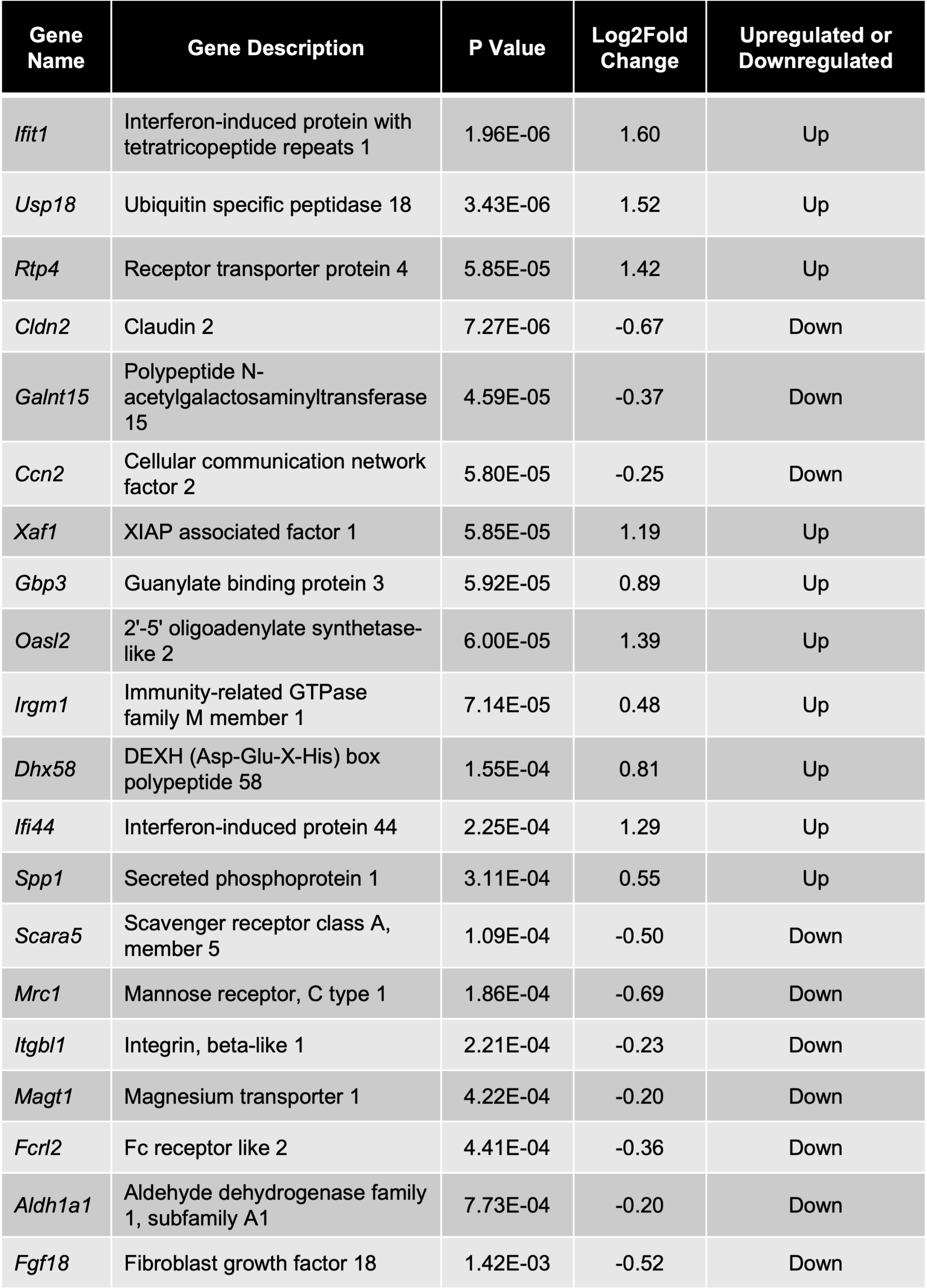

Supplement: S4 Table — Upregulated genes are more related to an immune and pro-inflammatory response. Downregulated genes are more involved in cellular communication. (TIF) [file ppat.1012733.s009.tif]
