# Supplementary material for: Toxoplasma gondii infection of neurons alters the production and content of extracellular vesicles directing astrocyte phenotype and contributing to the loss of GLT-1 in the infected brain
Source: PLoS Pathog. 2025 Jun 16;21(6):e1012733. doi: 10.1371/journal.ppat.1012733 (PMC12193631; doi:10.1371/journal.ppat.1012733)
Supplement: Attachment — Submitted filename: Tabaie_2025 FINAL RESUBMISSION Response to reviewers only.pdf [file ppat.1012733.s011.pdf]

E.H. WILSON PH.D
ASSOCIATE DEAN OF GRADUATE DIVISION
PROFESSOR OF BIOMEDICAL SCIENCES
UNIVERSITY OF CALIFORNIA, RIVERSIDE
emmaw@ucr.edu

We would like to thank all the reviewers for their valuable feedback and suggestions. We have substantially revised the manuscript. This includes additional western blot analysis of EV content, new imaging analysis of EV location, and scRNAseq analysis of astrocytes in vivo following infection, demonstrating similar transcriptional changes as induced by the addition of EVs *in vitro*. Of note we verify the EV location of GRA7 in astrocytes and observe GRA7 staining within the nucleus of EV containing astrocytes and not in EV negative cells within the same well, supporting the ability of Toxoplasma derived proteins to alter astrocyte responses via EV uptake.

This new data and all other requests and suggestions from the reviewers has significantly improved our study. Please find attached a point-by-point response to all comments and suggestions.

Reviewer #1:

The authors seek to explore how Toxoplasma infection influences the responses or characteristics of cells in the vicinity of the infected cell that may not themselves be infected. These studies were informed by previous studies that had found that secretions from neurons including secretion of extracellular vesicles (EVs) can modulate gene expression of astrocytes. The approach of the authors was to first characterize the effects of Toxoplasma infection on EVs that are released from Toxoplasma infected neurons as compared to EVs from uninfected neurons. Specifically, they assess the effects on infection on numbers of EVs released as well as on the content of their cargo (protein and nucleic acid (miRNA) content). They then proceed to evaluate the effects of these EVs on astrocytes by incubating EVs with astrocytes, after which they evaluate the gene expression of the astrocytes. They observed that EVs from Toxoplasma infected neurons have a modest effect on GLT protein levels in astrocytes through an uncertain mechanism. Overall, the findings are interesting and significant. However, below are suggestions that they should consider adding to their experimental approaches that could better clarify if not strength their findings.

We would like to thank reviewer 1 for summarizing our paper and being interested in our data. We appreciate the time and effort dedicated to carefully evaluating our work and offering suggestions for improvement to enhance our findings.

Major Issues

1. The evaluation of EVs from the neuronal primary cells must be challenging. As they report, neuronal cells are sensitive to the infection loaded, which necessitated that they perform experiments with a relatively low parasite: neuronal cell ratio. It would be informative to include the cell numbers (neurons) at the start of infection as it provides necessary information about the system.

We completely agree with the reviewer that we should have provided further information on the culture system, especially the number of cells before and after infection and the MOI. The seeding of embryonic cortical stem cells does not lead to 100% conversion to neurons. Indeed from 5 million plated cells ~10, 000 fully develop to cortical neurons. These counts were made by direct microscopic examination and did not require harvesting of cells thereby avoiding cell loss. Cell number remained the same for the length of the culture period and following infection. These new numbers reveal that the actual MOI is closer to 250:1. However, direct microscopic counts of infected versus uninfected neurons reveals that only 50% of neurons are infected. This may be explained by the reduction in surface area that neuronal cultures cover compared to more traditional confluent fibroblast culture and because uninvaded parasites are removed after 3 hours. Indeed, changing the MOI led to very few differences in the proportion of infected cells (**Supplemental Figure 1B**) suggesting we have achieved maximal neuronal infection.

We have now added further culture information to the manuscript in the methods, and results on **pages 6 (lines 138-140) and 23 (lines 639-643)** and believe it will help the interpretation of the results as well as the ability for others to use this culture system.

2. The authors should consider assessing the effect of inert particle uptake by these neuronal cells. If inert particles do not affect EV particle release, it should support their observation that the effect of Toxoplasma is physiologically distinct.

The reviewer brings up an interesting point suggesting a more general process of taking up particles by a neuron may lead to the reduction in EV production rather than the presence of the parasite. However, it is important to note that neuronal cells are not taking up parasitic particles but instead are actively invaded by Toxoplasma*.* During invasion the parasite attaches to the outer surface of the cell through the release of microneme proteins (MICs). A parasite specific structure, the moving junction, forms between host and parasite cell membranes to generate a parasitophorous vacuole membrane (PVM) isolating it from the host cell cytoplasm. Importantly, the formation of the PVM is segregated from the host endocytic and exocytic vesicle trafficking pathway: *Mordue et al 1999. Toxoplasma gondii resides in a vacuole that avoids fusion with host cell endocytic and exocytic vesicular trafficking pathways. Exp Parasitol. 1999 Jun;92(2):87–99*.

Thus, although the concept that EV release is reduced during other cellular functions is of interest, we do not believe that adding this control warrants the additional experiments due to the active and highly specific and very different process of parasite invasion.

3. They show the size of particles obtained from TEM. Although it is often used in the characterization of EVs, the TEM protocol results in dehydration of the samples, which results in significant shrinkage of EVs. They show that the average size from uninfected and infected samples is approximately 75 – 77nm. So, while the morphology might be informative, the size obtained from TEM isn't.

The authors agree with the reviewer that TEM can result in around a 12% shrinkage of the samples (*Van Der Pol E, et al. Particle size distribution of exosomes and microvesicles determined by transmission electron microscopy, flow cytometry, nanoparticle tracking analysis, and resistive pulse sensing. J Thromb Haemost. 2014 Jul;12(7):1182–92).* Although this reduction does not alter the conclusion, we have deemphasized the use of TEM to quantify the size of particles (changes within the text on **page 6, lines 153-154**).

4. The point just raised is amplified by the 'noisy' NTA profile of EVs in their preparations. Based on the NTA profiles, there appears to be a significant shift towards much smaller particles after Toxoplasma infection. There is ample information that EV isolation kits (protocols) can differentially enrich or exclude subpopulations of EVs. While CD63 is an accepted reproducible component of EVs, it would be informative if they performed Western blots where other accepted EV markers (TSG101, CD9 and others) are evaluated. Such analysis could provide a bit more insight on the underlying mechanism of Toxoplasma suppression of EV release.

We agree with the reviewer and have now added Western blot analysis for CD9 and Calnexin. This confirms the presence of CD9 in EV samples from uninfected and infected neurons and the absence of ER in all EV samples. This data is provided in new **Figure 2F** and addressed in the text on **page 7, lines 167-170**. It is also important to note that graphing EV size as measured by NTA reveals no significant difference following infection as now provided in the new **Figure 2D**.

5. The LC/MS/MS data in Figure 3 is fine. However, it is useful to provide validation of some kind that supports that those molecules are indeed present in their preparation. A Western blot showing a representative few of these identified molecules would suffice.

We have now added Western blots probing for mammalian Vimentin (Vim), Heat shock Protein 90 beta (HSP90B), and Toxoplasma GRA7 as seen in the new **Figure 3F** and agree this validates and therefore greatly improves our data. Reference to these new data and Figures can be found within the text on **page 8, lines 216-221**.

6. In the experiments in Figure 5, z series images are used to assert that EVs from neuronal cells are taken up by astrocytes. A more quantifiable approach would be to perform immunofluorescence to detect a known EV component (parasite derived molecule, for example) under permeabilized or non-permeabilized conditions. The compartment in which the molecule of interest is localized would less ambiguous.

Thank you for this suggestion. We have provided orthogonal x/z and z/y projections to better demonstrate the intracellular location of the EVs in **Supplemental Figure 3A**. We also conducted a permeabilization assay staining for GRA7, a parasite derived protein found within harvested EVs from infected neurons. In the absence of permeabilization no EVs were observed (data not shown) confirming their intracellular location. In addition, the cytochemistry for GRA7 was revealing by showing a) not all EVs were GRA7 positive and b) in cells that contained GRA7+ EVs, GRA7 was observed in the nucleus but was absent from the nuclei of EV negative cells. Altogether this supports the intracellular location of the EVs and the concept that the parasite derived content of these EVs has the potential to alter astrocyte activation. Lastly, the presence of GRA7 negative EVs and GRA7 containing EVs may favor the model of EVs being derived from both the host cell and the parasite cell rather than just the content. We'd like to thank the reviewer for their suggestion that led to this finding and John Boothroyd and Li-av Zarko for the generous donation of the GRA7 antibody and helpful discussions. The data is provided in new **Figure 5D** and **Supplemental Figure 3C** and discussed within the text on **pages 10 (lines 262-269) and 18 (lines 499-500)**.

7. In the experiments described in Figure 6, the PCA plots include gene expression after LPS/IFNy. There is no mention whether EVs from infected neurons impact the activation by LPS/IFNy

Thank you for this observation. We utilized LPS/IFNy as a positive control for astrocyte stimulation and to provide some scale for the transcriptional changes seen in astrocytes induced by EVs. This stimulation (presence of bacterial LPS) does not occur in Toxoplasma infection and therefore although there is interest in how potent EVs from neurons may be in this context, we focused our studies on the effect of Toxoplasma infection on communication between neurons and astrocytes. However, analysis of transcriptional changes induced by such an inflammatory stimulus can show the specificity of the signal provided by the EVs. We have now included this additional analysis (**Supplemental Figure 4**) that demonstrates the distinct signatures generated by LPS stimulation and stimulation by EVs from infected neurons and discuss this within the text on pages **13 (lines 354-356), 19 (lines 531-534) and 20 (lines 543-548).**

Minor Issues

8. Figure 4 legend needs to be clarified 'miRNAs after the addition of EVs from Uninfected EV and Infected EVs'. Were these EVs recovered from infected cells or were they added to infected cells?

We agree this wording was incorrect and have changed it to better indicate that the miRNA sequencing was conducted on EVs isolated from both uninfected and infected neurons in our Figure 4 legend.

Reviewer #2 (Comments to the Author):

In this study, Tabaie and colleagues characterize how infection of neurons with T. gondii cysts alters the quality of the extracellular vesicles released by neurons and look at their effects on astrocytes. The study addresses an important issue regarding how neuronal-astrocyte interactions may be altered, especially focusing on glutamate receptor expression on astrocytes, which may have an impact on glutamate homeostasis. The experimental approaches are primarily centered on in vitro cultures and modelling the interactions by co-culturing of neuron-derived EVs and primary astrocyte cultures. The study came up with potentially interesting observations, which need to be further validated. Overall, the study is still in a descriptive stage, but this may be of interest to the readership.

We would like to express our gratitude to the reviewer for their thoughtful comments and valuable insights provided during the review process of our manuscript.

Major Issues

1. As written, some sections of the Abstract may be misleading. For example, "EVs from infected neurons contained secreted Toxoplasma proteins GRA1, GRA2, GRA7, MAG1 and MAG2 associated with cyst formation"---- not all the proteins enumerated are necessarily cyst-specific. Also, "EVs from infected neurons altered gene expression--- leading to a downregulation of GLT-1 protein expression..". Here the word "leading" is not supported by the data, since we do not know how the EVs decreased GLT-1 protein but not mRNA levels.

We agree with the reviewer's comments on our misleading wording in the abstract. We have rewritten the abstract for clarity.

2. The most interesting part of the data set presented is in Figure 6, showing an IFN-like signature induced by EVs from infected neurons and decreased GLT-1 protein expression, but no suppression of its mRNA. There is also a group stimulated with LPS/IFNg, but this is not shown for the rest of the Figure. It would be important to show whether this strong stimulation combination also exhibits the same properties as the EVs from infected neurons, perhaps in magnified way. Importantly, it would be important to probe, whether blockade of interferon-signaling is required to observe the downregulation of GLT-1 protein. A functional assay to show the effect of the downregulation would be good to also include.

We agree the data in Figure 6 is especially interesting. As commented in our response to Reviewer 1 our use of LPS and IFN-g in these experiments was to provide a positive control for astrocyte activation and give some scale to the magnitude of the response. However, as the reviewer points out we can also use this stimulation to help determine the specificity of the astrocytic response. We have now included GO term analysis of transcriptional changes induced in astrocytes by LPS and IFN-g alongside those induced by EVs from infected neurons (**Supplemental Figure 4**). As the reviewer predicted, the magnitude of the response to LPS and IFN-g is far greater with up to 300 genes upregulated in some GO terms compared with a maximum of 80 genes upregulated by EVs from infected neurons.

Most importantly the signature of these transcriptional changes is quite different. The top GO terms in response to LPS and IFN-g are focused on generation of RNA and protein (ribonucleoprotein biogenesis) followed by 'regulation of innate immune response' and 'response to virus'. In comparison the dominant transcriptional signature in astrocytes incubated with EVs from infected neurons is about chemotaxis ('chemotaxis'; 'leukocyte migration').

The 'defense response to protozoan', a particularly striking finding when comparing EVs from infected versus uninfected neurons, is also an upregulated GO term in response to LPS-IFN-g. This should be expected with IFN-g a required signal for any immune response against protozoa. It is the specificity achieved by EVs from infected neuron cultures where there is no IFN-g present that makes our EV findings important. This new analysis is discussed on **pages 13 (lines 354-356), 19 (lines 531-534) and 20 (lines 543-548)**.

The reviewer also asks about the IFN-g regulation of GLT-1. Our previous work (*David et al, Plos Pathogens, 2016*) demonstrated significant downregulation of GLT1 and upregulation of extracellular glutamate concentrations *in vivo*, as measured by western blot, immunohistochemistry and *in vivo* microdialysis measurements followed by LCMS of extracellular fluid from the infected brain. This downregulation of GLT1 led to morphological and functional changes in neurons that could then be rescued by ceftriaxone-mediated upregulation of GLT-1. There is no lack of IFN-g in the brain, indeed fatal pathology occurs in its absence. Therefore, we do not think blocking IFN-g is required for the downregulation of GLT-1. Further in this current study cultures that have no IFN-g i.e. all our samples in Figure 6E do not show downregulation of GLT-1 unless stimulated with EVs from infected neurons.

We have added our LPS/IFNy stimulation group to our existing graph on *Slc1a2* normalized counts, and protein analysis (**Figure 6D-F**) to demonstrate that a proinflammatory signal is in itself not a cause of GLT-1 downregulation.

Reviewer #3 (Comments to the Author):

The manuscript from Tabaie, et al explores the impact of T. gondii infection of neurons on extracellular vesicles in vitro. The authors infect primary cortical neurons with T. gondii and examine the number and content of EVs derived from the neuronal cultures. They found that infected neurons produced fewer EVs and the EVs contain neuronal and parasite proteins. The neuronal EVs were then used to treat cultured astrocytes. The authors observed that astrocytes responded to the EVs from the infected cells and induced genes related to an immune response. Overall, the results suggest that infected neurons are altered and their EV content could impact glial function in the brain. The results may reflect processes in the infected brain, but this was not confirmed in the current manuscript.

We appreciate reviewer 3’s constructive feedback and believe their suggestions have significantly strengthened the quality and clarity of our manuscript.

Major Issues

1. To make a potential connection between the observations made in vitro to the brain during infection, the gene expression changes in the astrocytes in vitro could be made to the known changes in astrocyte gene expression in the brain in response to infection. The authors nicely tie their results back to GLT-1, but are the other genes reflected in vivo (response to virus, etc.)

The reviewer makes a very valuable point and although the translation of a highly reductive *in vitro* experiment to an *in vivo* infection with multiple, time-dependent cell-to-cell interactions is not trivial, we have spent some time analyzing astrocyte gene expression during Toxoplasma infection. We currently have an additional manuscript provisionally accepted at *Glia* (Figueroa et al 2025) that addresses astrocyte heterogeneity during Toxoplasma infection. Using this data set (publicly available GEO: GSE274474) we have analyzed the genes upregulated by astrocytes specifically in response to EV stimulation and find these expressed almost exclusively in astrocytes only from infected mice with transcriptional signatures that closely resemble the GO terms induced by EVs. This data and further analysis of 2 specific subpopulations are now presented in **Figure 7**. We’d like to thank the reviewer for pointing out this possibility and believe this additional information helps the impact of our study. These results are primarily discussed in the text on **pages 14 (lines 363-388) and 20 (lines 555-557)**.

2. A major conclusion is that infected neurons generate fewer EVs. While the authors confirm that there is a similar number of neurons in each culture, the neurons have an altered morphology and appear to be sicker, including condensed nuclei. Additional measures of neuronal health would be helpful in understanding the health of the neuron. The authors state that higher MOIs “overwhelm the cultures” and lead to neuronal damage. At the lower MOI, the cells may still be quite sick.

We agree with the reviewer that the health of the neuronal cultures is important. There are examples, that suggest Toxoplasma infection alters neuronal morphology and function most comprehensively from the Koshy group demonstrating the direct injection of parasite proteins leading to a significant decrease in firing potential and loss of these neurons over time (Mendez 2021). Alternatively, Toxoplasma secretes proteins preventing host cell death potentially allowing long term survival of the parasite, including in cyst form (Rosenberg and Sibley 2021) and minimizing pathology-causing cell death signals within the brain (Shallberg and Hunter, 2021).

Neuronal cultures are sensitive and cells die rapidly (<24hrs) under sub-optimal conditions. As previously described in the manuscript, care was taken to optimize these cultures to maximize the health of the neurons and collection of EVs over the course of 3 days of culture. This includes optimizing the MOI with an original parasite:neuron ratio of 0.5 leading to approximately 50% of neurons infected. In addition, we have provided analysis of NeuN and MAP2 cytochemistry and analysis of dendrite morphology, all measurements of neuronal health, **Figure 1D** and **Supplemental Figure 1C**. More than 95% of neurons were positive for NeuN and MAP2 whether or not they were infected demonstrating cell viability comparable across conditions.

3. In the PCA plot examining changes in gene expression in astrocytes, the authors compare the changes to cells treated with LPS and IFN-g. The inclusion of this treatment group was incredible useful to gauge the degree of the response to the EVs. The zoom in on the PCA plot is necessary to appreciate differences between the EV-treated groups. The authors should comment that the PCA plot does not show dramatic changes between the groups. The samples could also be re-analyzed without the LPS/IFN group to make the differences more obvious (or not).

We thank the reviewer for highlighting the use of the LPS-IFNg data and we realize we had not remarked on the closeness of the EV samples and have now done so. We have also re-analyzed the data without LPS/IFNγ and again without LPS/IFNγ and Media No Dex, **Supplemental Figure 4A-B**. Similar groupings between the different conditions were seen in the PCA even without LPS, validating the gene similarities between the replicates and the distinctions between the different conditions.

4. In several panels including Figure 1B, 1C, 5C-D, it is unclear what the "n" represents. For example, is each data point in the graph a single cell, single culture, or single experiment (with data averaged across wells). Clarity in the figure legend would be helpful to understand where the variation is coming from.

We thank the author for catching this discrepancy. We have clarified the replicate information in the methods and in all the figure legends.

Minor Issues

5. In the first line of the abstract, the authors state that neurons are the only cell type where cysts form, which is not entirely accurate. Later in the manuscript the authors state that the majority of cyst formation is in neurons. The abstract should be amended to be consistent with the latter statement. Also, the infection is likely not dormant for the lifetime of the host (particularly in mice).

We appreciate this comment and have corrected this inaccuracy.

6. Line 62-63 of the introduction needs a citation at a minimum. The statement that the parasite manipulates cell signaling pathways, inhibits host defense, etc. is all true but perhaps based on multiple cell types. Far less is known about how the parasite specifically affects neurons. The statement should be supported by citations and the cells types in which these findings were observed should be reflected.

We have amended this statement to include specific cell types with citations on **page 3 (lines 77-80)**.

7. Line 76. Could astrocyte-neuronal communication also be affected by secreted factors and not just secreted EVs?

Secreted factors do play a role in astrocyte-to-neuron communication. Astrocytes and neurons can release neurotransmitters, hormones, and neuromodulators, which are all able to alter this cell – cell communication. This concept has been expanded upon in **page 4 (lines 91-94)**.

8. Figure 4B would be improved by including the miRNA in the heatmap.

We have added the names of the miRNA to the heatmap in **Figure 4B**.
